# Surface step terrace tuned microstructures and dielectric properties of highly epitaxial CaCu_3_Ti_4_O_12_ thin films on vicinal LaAlO_3_ substrates

**DOI:** 10.1038/srep34683

**Published:** 2016-10-05

**Authors:** Guang Yao, Min Gao, Yanda Ji, Weizheng Liang, Lei Gao, Shengliang Zheng, You Wang, Bin Pang, Y. B. Chen, Huizhong Zeng, Handong Li, Zhiming Wang, Jingsong Liu, Chonglin Chen, Yuan Lin

**Affiliations:** 1 State Key Laboratory of Electronic Thin films and Integrated Devices, University of Electronic Science and Technology of China, Chengdu, Sichuan 610054, P. R. China; 2 Department of Materials Physics and Chemistry, Harbin Institute of Technology, Harbin 150001, P. R. China; 3 National Laboratory of Solid State Microstructures & Department of Materials Science and Engineering, Nanjing University, Nanjing 210093 China; 4 National Laboratory of Solid State Microstructures & Department of Physics, Nanjing University, Nanjing 210093 China; 5 State Key Laboratory Cultivation Base for Nonmetal Composites and Functional Materials, Southwest University of Science and Technology, Mianyang 621010, P. R. China; 6 Department of Physics and Astronomy, University of Texas at San Antonio, San Antonio, Texas 78249, USA; 7 Department of Physics and the Texas Center for Superconductivity, University of Houston, Houston, Texas 77204, USA

## Abstract

Controllable interfacial strain can manipulate the physical properties of epitaxial films and help understand the physical nature of the correlation between the properties and the atomic microstructures. By using a proper design of vicinal single-crystal substrate, the interface strain in epitaxial thin films can be well controlled by adjusting the miscut angle via a surface-step-terrace matching growth mode. Here, we demonstrate that LaAlO_3_ (LAO) substrates with various miscut angles of 1.0°, 2.75°, and 5.0° were used to tune the dielectric properties of epitaxial CaCu_3_Ti_4_O_12_ (CCTO) thin films. A model of coexistent compressive and tensile strained domains is proposed to understand the epitaxial nature. Our findings on the self-tuning of the compressive and tensile strained domain ratio along the interface depending on the miscut angle and the stress relaxation mechanism under this growth mode will open a new avenue to achieve CCTO films with high dielectric constant and low dielectric loss, which is critical for the design and integration of advanced heterostructures for high performance capacitance device applications.

Functional complex oxide thin films with controllable microstructures and physical properties are playing more and more important roles in microelectronic device development due to the increasing functional diversification in integrated circuits^1^,^2^,^3^,^4^. Perovskite-like CaCu_3_Ti_4_O_12_ (CCTO) with its ultra-high and stable dielectric constant over a broad frequency range from 0 Hz to 1 MHz in the temperature range of 100~600 K has been considered as a promising dielectric material candidate for various capacitance device applications^5^,^6^. The ultra-high dielectric constants of CCTO films are normally considered to be the results of the internal barrier layer capacitors (IBLC) consisting of semiconducting grains and insulating grain boundaries^7^,^8^,^9^,^10^,^11^. To further understand the nature of this material for practical device applications, it is necessary to systematically study the relationship between the physical properties and the atomic microstructures in high quality CCTO thin films with feasible ways to manipulate their physical properties. Strain engineering is always considered as a practical technique to tune the properties of various multifunctional oxide thin films. In the last decade, techniques with controllable strains in epitaxial thin films have been widely investigated^12^,^13^,^14^,^15^,^16^,^17^,^18^,^19^,^20^. Recently, the surface-step-terrace (SST) model was developed to tune the interface strain by controlling the unit cell matching between the film and the surface step terrace dimension of the substrate^21^,^22^,^23^,^24^. In this model, there are two kinds of mismatch between the film and the vicinal substrate: the lattice misfit between the film and the substrate and the unit cell mismatch between the epitaxial film and the surface step terrace dimension of the substrate. Both lattice misfit strain energy and unit cell mismatching strain energy will be stored in the epitaxial film. Thus, by adjusting the miscut angle of the vicinal substrate, the unit cell mismatching strain energy can be tuned by controlling the surface step terrace dimension. Therefore, the SST model provides a new way to tailor the strain in the epitaxial oxide thin films. Since the impact of strain on its dielectric properties is still lacking, it is worthy to systematically study the strain effect of CCTO thin films using the design based on the SST model, which would be a practical way to fully understand the mechanisms of the strain engineered physical properties in highly epitaxial CCTO films.

In this paper, we have successfully fabricated highly epitaxial CCTO thin films with the thickness of 25 nm on various vicinal single-crystal pseudocubic LaAlO_3_ (LAO) substrates by using a chemical solution deposition technique named polymer-assisted deposition (PAD)^25^,^26^,^27^. Correlations between the dielectric properties, strain behaviors, and the miscut angles of the substrates were carefully investigated. Based on SST matching growth mode, a model of coexistent compressive and tensile strained domains is proposed to understand the epitaxial nature.

## Results

**Figure 1:**
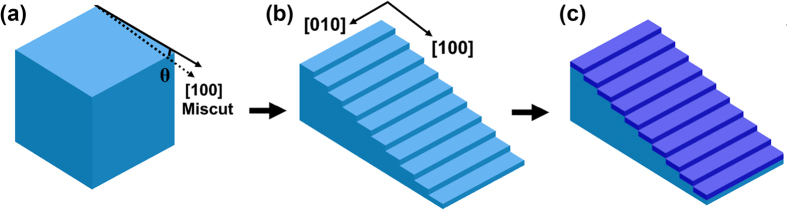
Flowchart describing the formation of epitaxial CCTO films on vicinal substrates. (**a**) The solid line with arrow shows the [100] direction of LAO substrate, and the dashed line with arrow shows the mechanically cutting direction of LAO substrate. *θ* is the miscut angle. (**b**) Miscut toward [100] produces nanosteps on LAO (001) surface with their edges along [010]. (**c**) The CCTO film grows along the nanosteps (the dark blue layer represent the CCTO film).

Experimentally, highly epitaxial CCTO thin films on vicinal (001) LAO substrates with miscut angles of 1.0°, 2.75°, and 5.0° along the [100] axis were designed and fabricated in this research. The schematic of vicinal LAO substrate was shown in Fig. 1(a). As a reference, a CCTO thin film on an ordinary LAO (001) substrate, i.e., a substrate with no intentional miscut, was also prepared. Ideally, vicinal substrates with the miscut along [100] can produce a vicinal surface with the step edge along [010], as schematically shown in Fig. 1(b). Therefore, the edges of films on vicinal substrates should grow straight along [010], as seen in Fig. 1(c). The sizes of the nanosteps will be determined by the miscut angle and the surface step terrace energy under different growth temperatures^28^,^29^.

**Figure 2:**
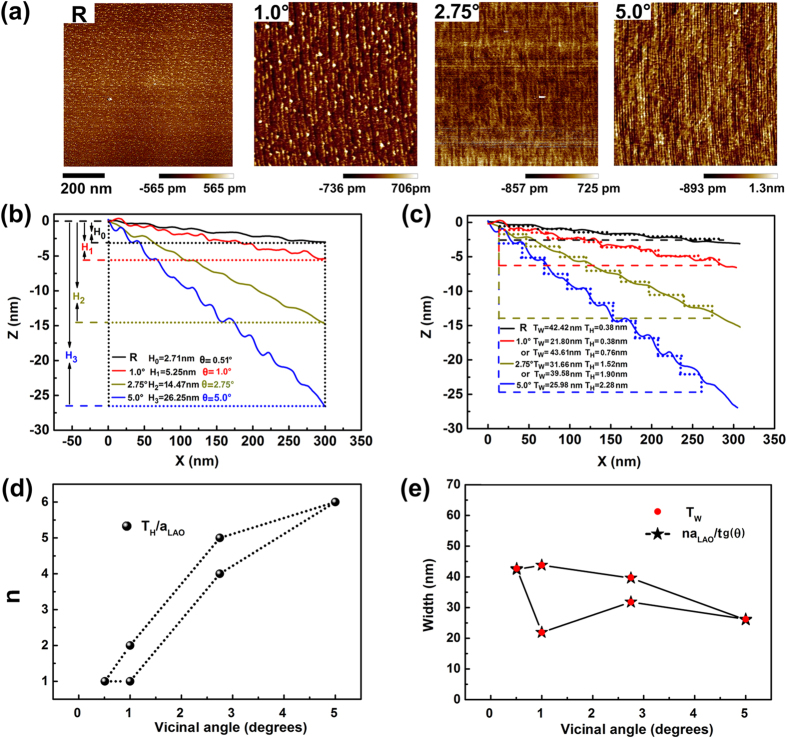
Morphologies of the vicinal substrates characterized by SPM. (**a**) SPM images of the reference and the vicinal substrates with various miscut angles of 1.0°, 2.75° and 5.0°. (**b**) Height profiles from the line scans on the selected sections of the vicinal LAO substrate. (**c**) Average width and height of each nanostep on various vicinal LAO substrates. (**d**) The number of LAO unit cells (*n*) corresponding to the average height (*T*_H_). (**e**) Comparison between the measured average width (*T*_W_) and the theoretical width based on trigonometric functions.

To figure out the actual step sizes under various miscut angles, scanning probe microscopy (SPM) was employed to characterize the morphologies of the vicinal LAO substrates after a thermal treatment of 240 minutes at 900 °C in the flowing O_2_ with a flow rate of 200 mL/min. The topography of the substrates is shown in Fig. 2. It is shown that the LAO substrates with the miscut along [100] can produce surfaces with step edge along [010], as shown in Fig. 2(a). Line scans across the selected 300-nm-wide sections on each vicinal LAO substrate are identified in Fig. 2(b). It should be noted that a specific miscut angle is calculated to be 0.51° for the ordinary reference substrate. As shown in Fig. 2(c), terraces produced on the surface of the LAO substrates can be well fit by a number of steps with average width (*T*_*W*_) and height (*T*_*H*_). It is indicated that the height of the steps are not always one unit cell of LAO. For example, for the 1.0° vicinal substrate, two kinds of steps with 1 and 2 unit cells in height respectively, were clearly seen to be present alternately. For the vicinal substrate with 2.75° miscut angle, average terrace height corresponding to 4.5 unit cells was detected, which can be considered to consist of two kinds of steps with 4 and 5 unit cells in height. The number of LAO unit cells (*n*) corresponding to the average terrace height (*T*_*H*_) is shown in Fig. 2(d), which are 1 and 6 for the substrates with miscut angles of 0.51° (the reference ordinary substrate) and 5.0°, respectively. Additionally, there are two adjacent steps with 1 and 2 unit cells height respectively for 1.0° vicinal substrate and two adjacent steps with 4 and 5 unit cells height respectively for 2.75° vicinal substrate. Theoretical width of step terraces can be calculated using the formula of *n* × *a*_LAO_/tg(*θ*), where *a*_LAO_ represents the lattice parameter of LAO and *θ* is the miscut angle. As shown in Fig. 2(e), the calculated values of terrace width are in good agreement with the measured values of average width (*T*_*W*_). The SPM results demonstrated that when the miscut angle is very small, step terraces with one unit cell in height can be obtained on the surface of the substrate, just like the reference substrate which processes a miscut angle of 0.51°. Since atoms at the edge of the steps have lower coordination than the ones in the middle, more step edges would increase the total surface energy. Thus, when the miscut angle increases to a certain value, a lower energy state is favored by step bunching, resulting in wider terraces separated by steps with *n* × *a*_LAO_ in height. Step bunching can be achieved by a high temperature treatment^30^. In other words, as the terrace dimension reduces with the increase of the miscut angle, the surface step terraces prefer to bunch together after the high temperature treatment. More work is ongoing to find out the basic correlation between the miscut angle and the terrace width.

**Figure 3:**
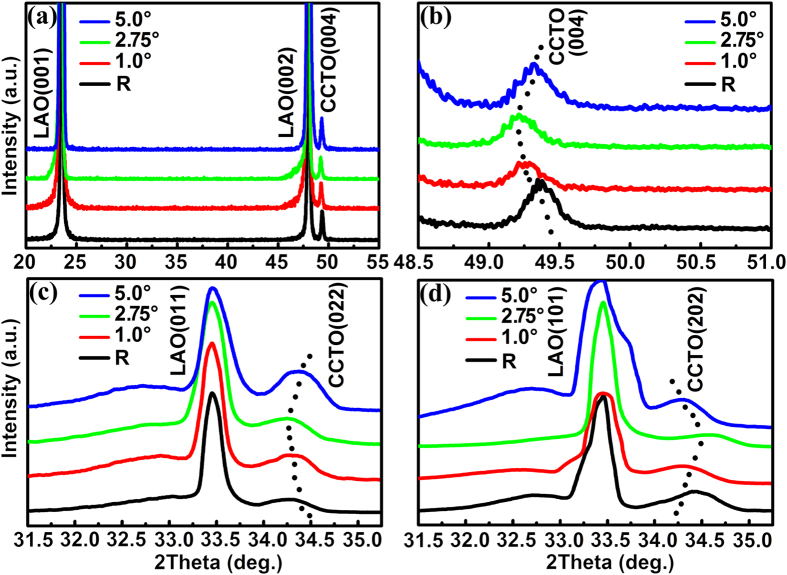
XRD *θ*–2*θ* scanning patterns of CCTO films grown on the reference and vicinal LAO substrates with miscut angles of 1.0°, 2.75°, 5.0°. (**a**) Normal (*χ* = 0°) XRD *θ*–2*θ* scanning patterns. (**b**) The fine normal XRD *θ*–2*θ* scanning patterns in a small range from 48.5° to 51.0°. (**c**) Tilted (*χ* = 45°) XRD scanning patterns from the diffraction of CCTO (022). (**d**) Tilted (*χ* = 45°) XRD scanning patterns from the diffraction of CCTO (202).

X-ray diffraction (XRD) was employed to examine the crystallinity and phases of the as-grown CCTO films. Especially, the strain status of the films, from both out-of-plane and in-plane lattice parameters, were analyzed through the normal (*χ* = 0°) and tilted (*χ* = 45°) *θ*–2*θ* scanning as well as reciprocal space mapping using high-resolution XRD. Figure 3(a) shows the normal (*χ* = 0°) XRD *θ*–2*θ* scanning patterns for all the samples. The sample on the reference substrate is also included in the figure for comparison. All the peaks in the patterns can be indexed by the (00*l*) diffractions from the perovskite-like CCTO and pseudocubic LAO, suggesting that the CCTO films were well fabricated with the preferred (00*l*) orientation on the substrates. To determine the interface strains, the diffraction patterns around the CCTO (004) peaks for all the samples were carefully rescanned and plotted in Fig. 3(b). It is observed that the CCTO (004) peaks are slightly shifted to lower angles when miscut angles of the substrates increases to 2.75°, suggesting that the *c*-axis lattice parameters of the films are stretched gradually. However, when the miscut angle further increases to 5.0°, the position of CCTO (004) peak moves back to an angle similar to that of the reference sample. The in-plane lattice parameters can be measured from the tilted XRD *θ*–2*θ* scanning technique (simply by tilting *χ* to 45°). To gain the information along [100] (*a*-axis) and [010] (*b*-axis), the incident X-ray was set to align to the two in-plane orientations in sequence, i.e., *ϕ* = 0° and *ϕ* = 90°. The as-measured XRD patterns from the diffractions of CCTO (022) and (202) for samples are shown in Fig. 3(c,d), respectively. From the figures, we can know that the peak shifts of both CCTO (022) and CCTO (202) show zigzag-like trends but their trends are opposite, suggesting anisotropic behavior in the evolution of in-plane strains for this set of samples. Thus, the lattice parameters for both in-plane and out-of-plane can be determined by carefully analyzing the normal and tilted scanning spectra, which were extracted and will be discussed later. Obviously, the largest peak shift in both normal and tilted XRD scanning spectra are from the film deposited on the substrate with the miscut angle of 2.75°.

**Figure 4:**
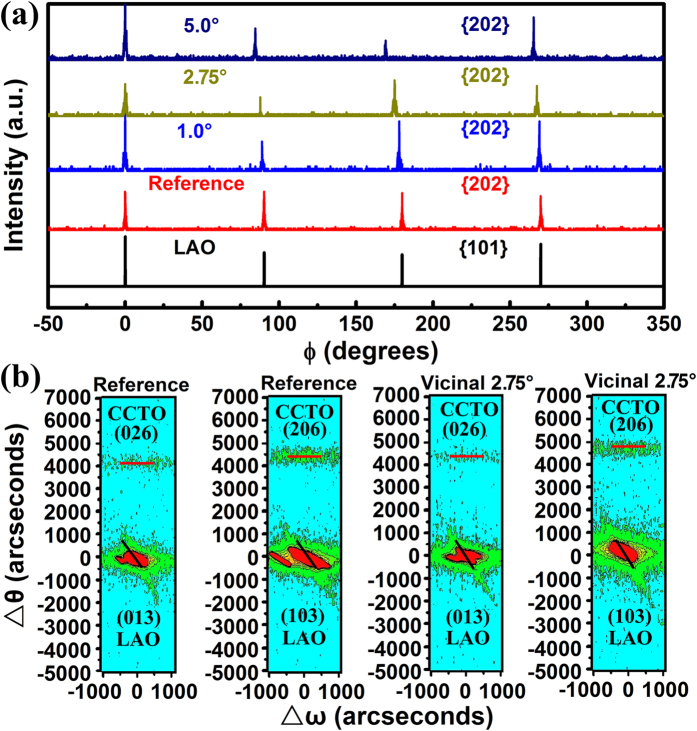
(**a**) The XRD *ϕ*-scanning patterns from {101} reflections of LAO substrates and {202} reflections of all CCTO films. (**b**) Asymmetric RSMs around the reflections of LAO (013)/CCTO (026) and LAO (103)/CCTO (206) for the reference sample and the CCTO film grown on the vicinal LAO substrate with 2.75° miscut angle.

Figure 4(a) shows typical XRD *ϕ*-scanning patterns from CCTO {202} and LAO {101} for samples grown on vicinal substrates and the reference sample, respectively. The good alignment between the four peaks from the films and the substrates demonstrates that the films are all with good epitaxial quality. The epitaxial relationship is determined to be (001) CCTO||(001) LAO and [100] CCTO||[100] LAO for all the samples. The average full width at half maximum (FWHM) of the peaks from the XRD *ϕ*-scans are 0.97°, 0.88°, 1.08° and 0.67° for the films on the reference, 1.0°, 2.75° and 5.0° vicinal substrates, respectively. The FWHM of the *ϕ*-scanning patterns can reflect the density of edge dislocations in the films^31^,^32^,^33^,^34^, which will be discussed later.

To further analyze the crystallinity and the epitaxial nature of the as-grown films, high-resolution XRD reciprocal space maps (RSMs) were collected to provide more details of the strain status of the films. RSM patterns around the (026) and (206) reflections of CCTO were measured by setting *ϕ* angle at 0° and 90°, respectively. Figure 4(b) shows the corresponding RSMs for the reference sample and the film grown on the 2.75° miscut substrate, respectively. Compared to the reference sample, a bigger Δ*θ* (difference between the diffraction spots of the CCTO and LAO) can be seen for the film grown on the 2.75° miscut substrate, no matter the *ϕ* angle was set at 0° or 90°, suggesting smaller lattice parameters along both in-plane directions of the film on the 2.75° miscut substrate. Moreover, more obvious anisotropy can be observed in the film on the 2.75° vicinal substrate compared to the reference samples, i.e., the difference between the Δ*θ* value of CCTO (026) and CCTO (206) is bigger for the former. The results are well consistent with those from the tilted (*χ* = 45°) XRD *θ*–2*θ* scans.

**Figure 5:**
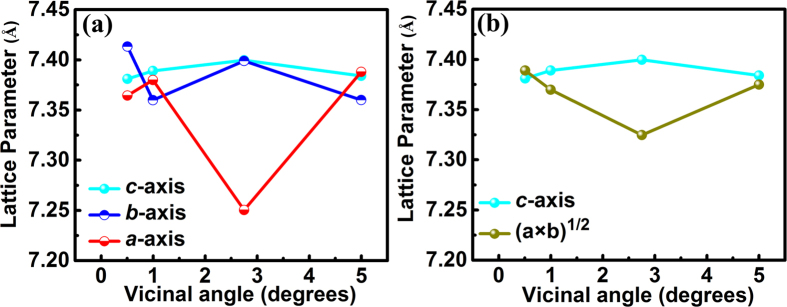
(**a**) Lattice parameters of the as-prepared CCTO thin films on the vicinal substrates with different miscut angles. (**b**) The *c*-axis and average in-plane lattice parameters of the as-prepared samples versus the vicinal angles of the substrates.

The out-of-plane and in-plane lattice parameters of the as-grown CCTO films extracted from Fig. 3 are shown in Fig. 5. Obviously, with the increase of the miscut angle, we see different strain evolution behavior along the two in-plane directions [010] and [100] in the samples. This should be attributed to the miscut along [100], which has induced different strain states parallel or perpendicular to the terrace steps. It should also be noted that the lattice parameters of [010] and [100] are not exactly the same for the reference sample. On one hand, this may be attributed to the complicated intrinsic domain structures of the pseudocubic LaAlO_3_ substrate^35^,^36^. On the other hand, although the reference substrate has not been intentionally miscut, a specific miscut angle of 0.51° was detected based on the SPM results (Fig. 2), which would induce strain in one direction and lead to the anisotropic in-plane lattice parameters based on the proposed model. To further evaluate the in-plane and out-of-plane strain states and their contribution to the change of cell volume respectively, we define an average in-plane lattice parameter as (*a* × *b*)^1/2^ (*a* and *b* represent the lattice parameters of [100] and [010], respectively) to minimize the effect from the variance in parameters of [010] and [100], as plotted in Fig. 5(b). The values of the average in-plane and the out-of-plane lattice parameters are very close to each other for the reference sample. Suggesting the stress from lattice mismatch and thermal mismatch has almost been fully released in this sample. However, it is clearly shown that the CCTO films grown on vicinal substrates bear in-plain compressive strain and out-of-plane tensile strain. The in-plane compressive strain increases with the miscut angle from 0.51° to 2.75°. But when the miscut angle increases to 5.0°, the strain is released resulting in similar average in-plane and out-of-plane parameters with the reference sample. The lattice of the CCTO film grown on the substrate with 2.75° miscut angle shows the largest deformation.

**Figure 6:**
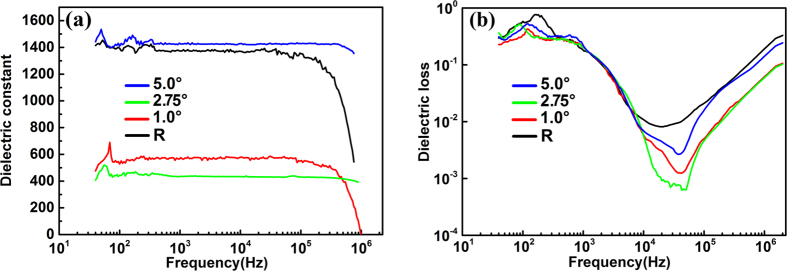
Dielectric constants (**a**) and loss (**b**) of the as-prepared CCTO films on the vicinal substrates with different miscut angles.

The dielectric properties from 40 Hz to 2 MHz are shown in Fig. 6. The evolution trend of dielectric constant with the miscut angle is very consistent to the trend of the average in-plane lattice parameter shown in Fig. 5(b), implying the strong impact from the in-plane strain on the dielectric constant of CCTO thin films. Additionally, at the frequency ranging from 10 kHz to 2 MHz, it is found that the values of dielectric loss for CCTO films on any vicinal substrates are lower than that for the reference sample. These results demonstrated that the dielectric constant can be tuned and the dielectric loss can be reduced by utilizing the SST growth mode.

## Discussion

**Figure 7:**
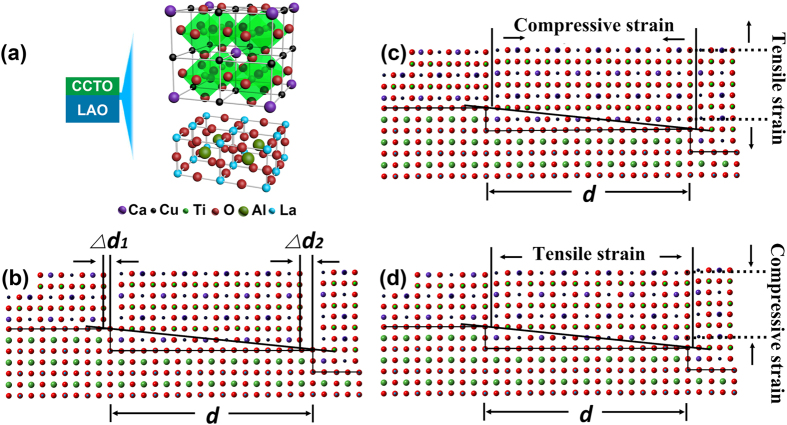
Schematic diagram showing atomic arrangement of the CCTO film on surface step terrace of the vicinal (001) LAO substrate. (**a**) Crystal structures of LAO and CCTO. (**b**) The generation of the “residual mismatching gap” at the end of the step terrace. (**c**) The formation of domain with the compressive strain along the interface and the tensile strain along the out-of-plane direction. (**d**) The formation of domain with the tensile strain along the interface and the compressive strain along the out-of-plane direction.

To understand the mechanism of the lattice deformation of CCTO thin films grown on vicinal substrates, mismatching strains introduced by surface step terraces should be taken into consideration^21^,^22^,^23^,^24^. As demonstrated by the SPM images in Fig. 2, the surface of the vicinal LaAlO_3_ substrate consists of surface terraces. The surface-step-terrace width of a LAO substrate cannot exactly accommodate integer unit cells or atomic planes of the CCTO film. Because of the ionic bonding of oxide, unlike metal or semiconductor, oxide thin film growth requires a good match in the combination of positive and negative charge balance. Theoretically, due to the difference in the total length of integer unit cells accommodated on the terrace between the epitaxial film and the substrate, the “SST residual matching gap” (Δ*d*) will be generated at each edge of the LAO step terrace. Practically, atoms of the CCTO film would rearrange on the surface step terrace to occupy the entire width of each terrace step, introducing “SST strain” in the film. The “SST model” is schematically shown in Fig. 7. Although the real scenario may be more complicated since the surface of LaAlO_3_ (001) may terminate in either LaO- or AlO_2_- plane depending on the preparation conditions^30^,^36^,^37^,^38^,^39^,^40^,^41^, the possibility of a termination with the mixture of two on the miscut LAO (001) surface has been reported to be very low^40^,^41^. Thus, we simply use one type of termination in Fig. 7 to demonstrate the basic idea of the SST matching model. To evaluate the SST strains in the as-prepared CCTO films, the terrace mismatching data for CCTO and vicinal LAO substrates with different miscut angles are calculated based on the lattice parameters of CCTO and LAO. It should be noted that since the domain matching happens at the growth stage, the lattice parameters of CCTO and pseudocubic LAO at the growth temperature (900 °C, *a*_LAO_ = 0.3823 nm, *a*_CCTO_ = 0.7422 nm), which are deduced by using the thermal expansion coefficients and the room temperature lattice parameters, should be used for the calculation^42^,^43^.

**Figure 8:**
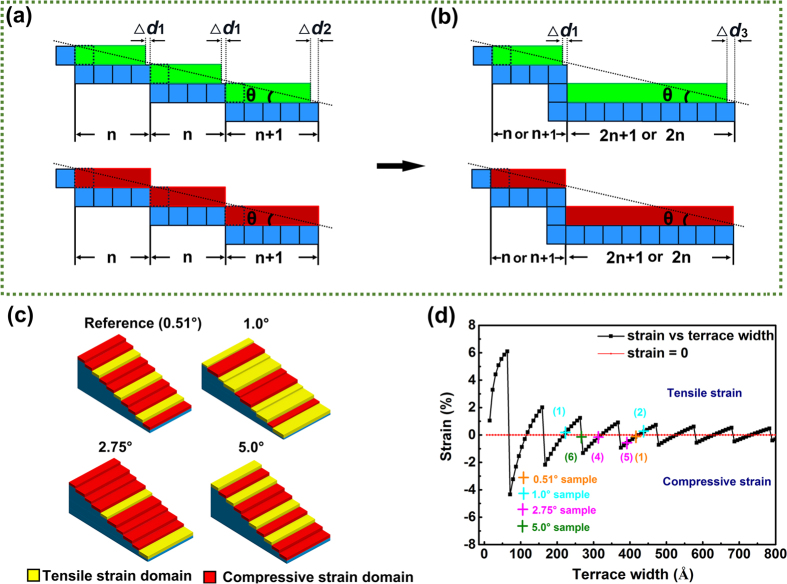
Schematic diagram showing the strain distribution in the CCTO films. (**a**) The schematic growth mode based on the step terrace with one unit cell in height for a vicinal substrate. (**b**) The growth mode on step terraces of a vicinal substrates after step bunching. (**c**) The non-uniform strain distribution in the CCTO films on vicinal substrates. (**d**) Surface-step-terrace-induced strain *δ* = (*a*_*f * −_ *a*_*bf*_)/*a*_*bf*_ of the film grown on vicinal substrates with different terrace width.

Since the terrace width may not be divided exactly by the lattice parameter of the LAO substrate while the crystal structure of the substrate would not deform to fit the terrace, the quantity of LAO unit cells accommodated on each terrace should be one of the two adjacent integers, as schematically shown in Fig. 8(a). For example, for the 1.0° vicinal substrate with 1 LAO unit cell in height, the terrace width divided by *a*_LAO_ is 57.29. Thus, there should be 57 LAO unit cells accommodated on one step for about 2/3 of terrace steps but 58 unit cells for the rest to ensure the dimension size of the LAO crystal. The surface step terrace prefers to bunch together upon annealing, and as shown in Fig. 8(b), it is true that terraces with different widths co-exist on the surface. On the other hand, for the CCTO film, as discussed above, the crystal would be strained to fit the terrace and eliminate “the residual matching gap”. Table 1 gives the width of the surface terraces (*T*_*w*_) of the vicinal substrates and the quantities of unit cells accommodated on each terrace for the substrates and the films, calculated based on the above-mentioned principles. The SST strains for all the cases were also calculated and listed in this table. The results suggested that the stress is not uniform in the films. In each CCTO film on the vicinal substrate, along the [100] direction, both compressive and tensile strains exist locally in the film. As shown in Table 1 and schematically indicated in Fig. 8(c), various miscut angles induce different percentages of CCTO compressive and tensile strained domains. For the CCTO film grown on flat LAO substrate, the lattice misfit would induce tensile strain in the film. However, according to the SPM results, the reference substrate has a specific unintentional miscut angle of 0.51°. Based on the proposed model, 68% steps bare compressive strain in one direction, leading to the global anisotropic in-plane strain. For the film grown on the substrate with 1.0° miscut angle, which processes two kinds of terrace steps (1 or 2 LAO unit cells in height), a higher percentage of the steps bare tensile strain, leading to an overall tensile strain along the [100] direction of the film. On the contrary, most of the steps bare compressive strain for the film grown on the substrate with 2.75° miscut angle which processes two kinds of terrace steps (4 or 5 LAO unit cells in height), resulting in a high global compressive strain along the [100] direction of the film. For the film grown on the substrate with 5.0° miscut angle, the numbers of the steps baring tensile strain or compressive strain are close to each other, which would minimize the overall internal strain along the [100] direction of the film. These calculated results agree very well with the [100] lattice parameters deduced from the XRD measurements which were shown in Fig. 5(a), implying that the SST strains have been successfully induced in the [100] direction of the CCTO films and dominate the strain states along this direction. To minimize the total energy of the system, the other in-plane axis, i.e. [010] axis, would be strained contrarily since there is no regulation from the terraces along this direction. The final resulted average in-plane strain and out-of-plane strain, as shown in Fig. 5(b), would be determined by the lattice mismatch, thermal mismatch, the SST strains, strain relaxation rate, as well as the Poisson ratio.

The method to predict the strain state on terrace steps has been reported earlier^21^. As shown in Fig. 7, “SST residual matching gap” (Δ*d*) will be generated at each edge of the LaAlO_3_ step. One more or less CaCu_3_Ti_4_O_12_ unit cell may deposit on a step terrace forming an in-plane tensile or compressive strain region to fit the terrace width, respectively. Surface-step-terrace-induced strain is defined as *δ* = (*a*_*f*_ − *a*_*bf*_)/*a*_*bf*_. The calculated strain versus terrace width is shown in Fig. 8(d). The red line (strain = 0) separates the tensile strain region and compressive region. The crosses label the corresponding strains of the CCTO films grown on the reference (0.51°), 1.0°, 2.75° and 5.0°, respectively. According to mismatch strain formula *δ* = Δ*d*/*d*, the stored strain decreases with the step dimensions widened. More experiments to further study the relationship between the miscut angles, terrace dimensions and preparation conditions are ongoing to improve this model.

The dielectric properties of the films on vicinal substrates are closely related to the strain behavior. It is found that the dielectric loss values of CCTO films on any vicinal substrates are lower than that of the reference sample at the frequency ranging from 10 kHz to 1 MHz. This may be related to the different strain relaxation mechanisms and the final in-plane strain states of the films on vicinal and normal substrates. For the CCTO film grown on the flat substrate, creation of edge dislocations would be a main route to release the stress from lattice or thermal mismatch. On the other hand, for the films on the vicinal substrates, the co-existence of domains with opposite strain states in the film would help release part of mismatching strain and thus reduce the density of dislocations. Moreover, with the increase of the miscut angle, the terrace dimension reduces and the density of the complementary strained domains increases, which would be more favorable to release the stress and reduce the density of edge dislocations. This effect would be obvious in the film grown on the 5.0° miscut substrate, since the percentages of the domains with compressive and tensile strains are almost equal in this film and the step dimension is the smallest among the samples. The lower density of edge dislocations can be proved by the narrower FWHM of the XRD *ϕ*-scanning peaks for the film on the 5.0° miscut substrate (0.67°) than the reference sample (0.97°). Reduction of the dislocations in the films would contribute to the decrease of dielectric loss. However, this effect may be negligible in the CCTO film on the 2.75° miscut substrate, as indicated by its FWHM of the XRD *ϕ*-scanning peak, since most of the steps bare compressive strain for this film. Thus, the contribution from the average in-plane strain shown in Fig. 5(b) should be considered. Our previous research has demonstrated that the in-plane compressive strain would help lower the dielectric loss of CCTO thin films^44^, which may play a key role in the film on the 2.75° miscut substrate. For the film on the 1.0° miscut substrate, both dislocation effect and the in-plane compressive strain effect may account for the reduction of the dielectric loss. On the other hand, the in-plane strain would also impact the dielectric constants of the CCTO films, as proved in our previous research^44^. As shown in Fig. 6(a), CCTO films on both substrates with 1.0° and 2.75° miscut angles exhibit lower values of dielectric constants compared to the reference sample, which should be related to their compressive average in-plane strain. However, for the film on the 5.0° miscut substrate, the dielectric constant is very close to that of the reference sample, since the stress in this film has almost been fully released. The evolution trend of dielectric constant with the miscut angle is very consistent to the trend of the average in-plane lattice parameter shown in Fig. 5(b), implying the strong impact from the in-plane strains on the dielectric constants of CCTO thin films. The results demonstrated that the dielectric loss can be reduced and the dielectric constant can be tuned by utilizing the SST growth mode. In the range of 10 kHz to 100 kHz, the film grown on the 5.0° vicinal substrate exhibits lower dielectric loss with a reduction by half an order of magnitude while maintains a similar dielectric constant, compared to the reference sample.

To figure out change tendency of physical properties with the thicknesses of films, CCTO films with different thicknesses (25 nm, 65 nm and 108 nm) were prepared. The results show that the effect of vicinal substrates on the strains of CCTO films is attenuated in a thicker film. The modulation on the dielectric properties of CCTO films by the vicinal substrates also becomes weaker with the increase of the thickness, which should be attributed to the strain relaxation. The details of thickness dependence are discussed in the Supplementary Information.

In conclusion, highly epitaxial CCTO thin films were grown on various vicinal (001) LaAlO_3_ substrates by using a polymer assisted deposition technique. The evolution of the CCTO lattice parameters along different orientations with different miscut angles indicated that the SST strains were induced in the films. The model of matching the surface step terraces with coexistent compressive and tensile strained domains in the films was proposed to understand the epitaxial nature. The overall strain states can be tuned by the ratio of the compressive and tensile strained domains, which is determined by the miscut angle. Therefore, the dielectric loss can be minimized and the dielectric constant can be manipulated by utilizing the proposed growth mode. The reduction of interface edge dislocation density due to the co-existence of the compressive and tensile strained domains as well as the final overall in-plane compressive strain induced by terrace step matching are considered to be the key roles to reduce the dielectric loss and tune the dielectric constant of the CCTO film.

## Methods

### Samples preparation

Epitaxial CaCu_3_Ti_4_O_12_ (CCTO) thin films were grown on normal and vicinal (001) single-crystal LaAlO_3_ (LAO) substrates with various miscut angles of 1.0°, 2.75°, and 5.0° by a polymer assisted deposition (PAD) technique. All the LAO substrates were ultrasonically cleaned in acetone, alcohol, and deionized water sequentially and annealed at 900 °C for 120 minutes in the flowing oxygen in order to form clean and ordered surface structures.

To deposit CCTO thin films by using the PAD technique, precursor solutions with the Ca^2+^, Cu^2+^ and Ti^4+^ ions were prepared, as previously reported^26^. Ca^2+^ and Cu^2+^ solutions were made by adding 1.68 g calcium nitrate and 1.93 g copper nitrate respectively into the solutions in which 3 g ethylenediaminetetraacetic acid (EDTA) and 3 g polyethyleneimine (PEI) were dissolved in 40 mL deionized water. The solutions were then purified and concentrated in an Amicon filtration unit to yield two solutions with the concentrations of 291.6 mmol Ca^2+^ and 395.1 mmol Cu^2+^ per liter, respectively. To prepare the Ti^4+^ solution, we slowly added 2 mL of titanium chloride into 30 mL 30% peroxide solution. After that, the solution was added slowly into the mixture of EDTA and PEI solutions. The resulting solution was purified and concentrated to get a solution containing 311.8 mmol Ti^4+^ ions per liter. The concentrations of the solutions were all measured by inductively coupled plasma optical emission spectrometer (ICP-OES). Eventually, the Ca^2+^, Cu^2+^ and Ti^4+^ solutions were mixed in the proportion of 1:3:4 to yield a homogenous precursor solution for CCTO. The as-prepared precursor solution was spin-coated on the substrates with a spin rate of 3000 revolutions per minute for 30 seconds. The samples were then put into a furnace for heat treatment. Briefly, the whole heat treatment process can be described as the following. To depolymerize the polymer, all the samples were first heated to 510 °C slowly and maintained at that temperature for 120 minutes. Then the samples were annealed at 900 °C for 120 minutes for crystallization. After that, all samples were cooled down naturally to room temperature. Flowing O_2_ with a flow rate of 200 mL/min was used during the entire heat treatment process. The thicknesses of the samples were checked by field emission scanning electron microscope (FESEM). (The cross-section image of the sample is shown in Fig. S1). The typical thickness of film is about 25 nm for all the samples.

### Microstructure measurements

Scanning probe microscopy (SPM) (Bruker, Multimode Nanoscope IIIa) and a commercial probe NSG 10 (NT-MDT) with a force constant of 20–80 Nm were employed to characterize the morphologies of the annealed LaAlO_3_ substrates. X-ray diffraction (XRD) including normal (*χ* = 0°) and tilted (*χ* = 45°) *θ*–2*θ* scanning, *ϕ*-scanning and reciprocal space mapping were conducted using a Bede D1 XRD system with Cu K_α_ radiation (*λ* = 1.54060 Å).

### Dielectric properties measurements

The dielectric properties of the as-grown CCTO films were characterized by using the interdigital capacitor technique^45^. As schematically shown in Fig. S2, the interdigital capacitor electrodes were prepared by using Au/Ni electrodes with a cross-finger-shape. A set of electrodes were prepared by lithography and dc-sputtering techniques. Each electrode pattern possesses a total of 100 fingers with the finger length of 190 μm, the finger width of 10 μm, and the finger gap of 10 μm. The capacitance and loss tangent of the samples were measured with the frequencies from 40 Hz to 2 MHz by using an Agilent 4294A Precision Impedance Analyzer.

## Additional Information

**How to cite this article**: Yao, G. *et al*. Surface step terrace tuned microstructures and dielectric properties of highly epitaxial CaCu_3_Ti_4_O_12_ thin films on vicinal LaAlO_3_ substrates. *Sci. Rep.* **6**, 34683; doi: 10.1038/srep34683 (2016).

## Supplementary Material

Supplementary Information

## Figures and Tables

**Table 1:** Terrace width (*T*_*w*_) of the vicinal substrates and the quantities of unit cells accommodated on each terrace for the substrates (*n*_s_) and the films (*n*_f_).

Sample	*T*_w_(nm)	*T*_w_/*a*_s_	*n*_s_	*a*_s_*n_s_(nm)	(*a*_s_*n_s_)/*a*_f_	*n*_f_	*a*_f_*n_f_(nm)	Stress	Percentage
0.51° off	42.70	111.68	111	42.44	57.18	57	42.31	Tensile	32%
			112	42.82	57.69	58	43.05	Compressive	68%
1.0° off (1- unit-cell- high)	21.90	57.29	57	21.79	29.36	29	21.53	Tensile	71%
			58	22.17	29.88	30	22.27	Compressive	29%
1.0° off (2- unit-cell- high)	43.80	114.60	114	43.58	58.72	59	43.79	Compressive	40%
			115	43.96	59.24	59	43.79	Tensile	60%
2.75° off (4 unit-cell- high)	31.84	83.28	83	31.73	42.75	43	31.91	Compressive	72%
			84	32.11	43.27	43	31.91	Tensile	28%
2.75° off (5 unit-cell- high)	39.80	104.09	104	39.76	53.57	54	40.07	Compressive	91%
			105	40.14	54.08	54	40.07	Tensile	9%
5.0° off	26.22	68.58	69	26.38	35.54	36	26.72	Compressive	58%
			68	26.00	35.02	35	25.98	Tensile	42%

The percentage of tensile strained domains and compressive strained domains are also calculated.
