# Supplementary material for: Surface step terrace tuned microstructures and dielectric properties of highly epitaxial CaCu3Ti4O12 thin films on vicinal LaAlO3 substrates
Source: Sci Rep. 2016 Oct 5;6:34683. doi: 10.1038/srep34683 (PMC5050425; doi:10.1038/srep34683)
Supplement: Supplementary Information [file srep34683-s1.pdf]

Surface step terrace tuned microstructures and dielectric properties of highly epitaxial $CaCu_3Ti_4O_{12}$ thin films on vicinal $LaAlO_3$ substrates

Guang Yao,[1)] Min Gao,[1)] Yanda Ji,[1)] Weizheng Liang,[1)] Lei Gao,[1)] Shengliang Zheng,[2)] You Wang,[2)] Bin Pang,[3)] Y. B. Chen,[4)] Huizhong Zeng,[1)] Handong Li,[1)] Zhiming Wang,[1)] Jingsong Liu,[5)] Chonglin Chen,[6),7)] Yuan Lin,[1)*]

Supplementary information

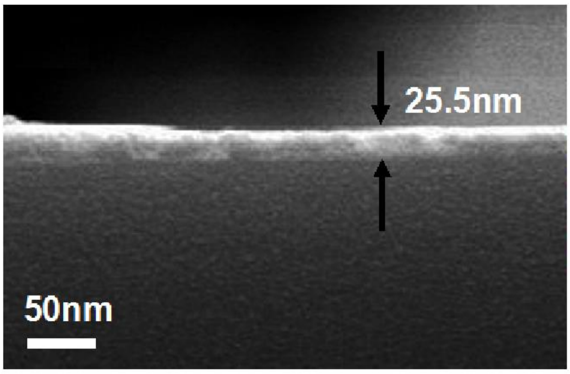

Figure S1. A typical cross-section image of the sample checked by field emission scanning electron microscope (FESEM).

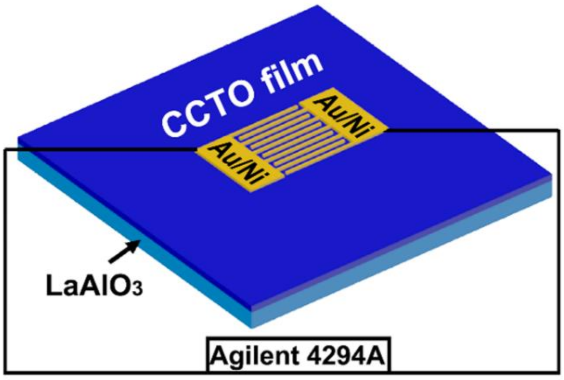

Figure S2. Measurement schematic of the dielectric properties for CCTO films.

To figure out change tendency of physical properties of CCTO films with the thickness of film, different thicknesses (25 nm, 65 nm and 108 nm) were prepared. As shown in Fig. S3, X-ray diffraction (XRD) was employed to characterize the strain states. The peak positions of CCTO (004) shift very little for the 65-nm-thick and 108-nm-thick films, indicating that the effects of vicinal substrates on the films are attenuated with the increase of the thickness.

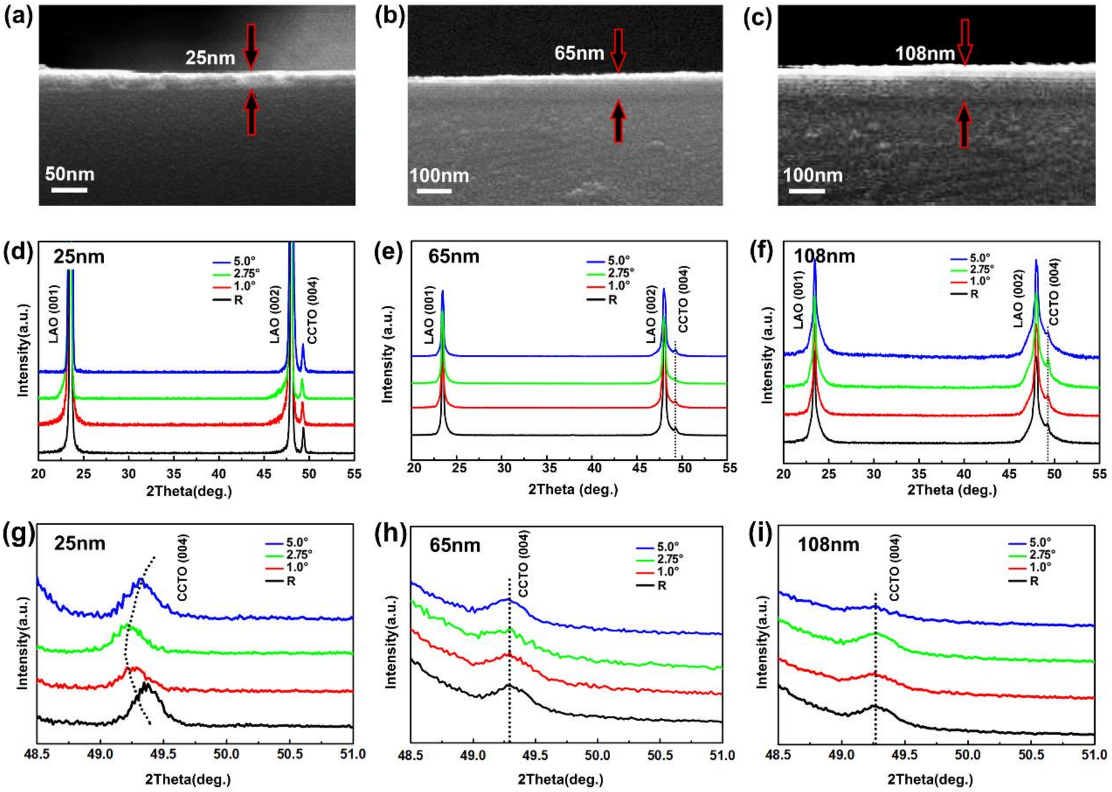

Figure S3. (a), (b) and (c) are cross-sectional SEM images of samples with different thicknesses. (d), (e) and (f) are X-ray diffraction patterns of samples with different thicknesses in a wide range from 20° to 55°. (g), (h) and (i) display the CCTO (004) peaks of samples with different thicknesses in a small range from 48.5° to 51.0°.

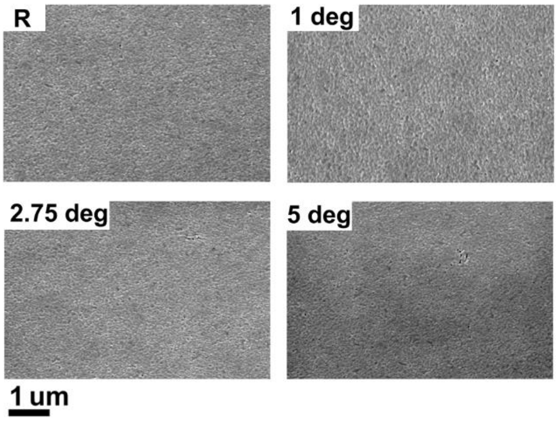

Figure S4. Surface morphologies of 25-nm-thick CCTO films with grown on vicinal substrates with different miscut angles.

As shown in Figure S4, no obvious difference can be seen between samples on substrates with different miscut angles.

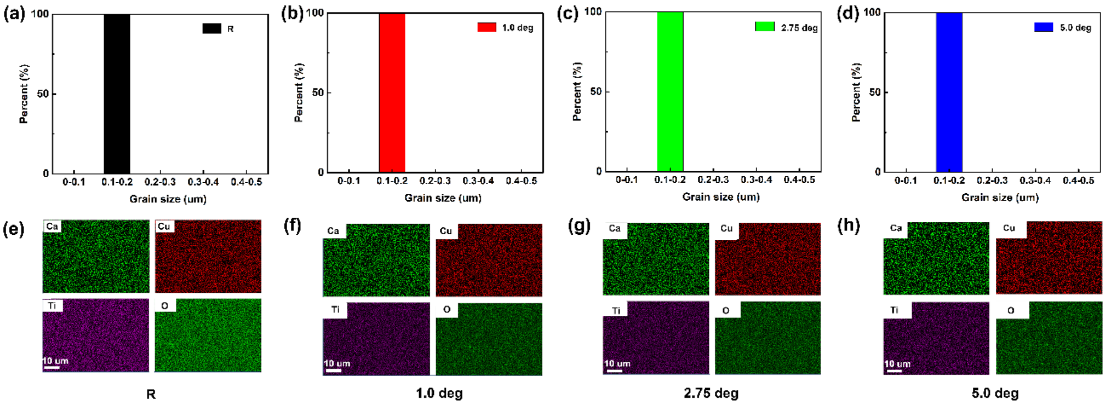

Figure S5. (a), (b), (c) and (d) are grain size statistics of 25-nm-thick CCTO films grown on vicinal LAO substrates with different miscut angles. (e), (f), (g) and (h) are energy dispersive spectrometer (EDS) maps of Ca, Cu, Ti and O of 25 nm CCTO films grown on vicinal LAO substrates with different miscut angles.

As shown in Fig. S5, grain sizes were measured for films and all grain sizes are in the range of 100 nm – 200 nm. Moreover, energy dispersive spectrometer (EDS) maps further confirm that the films are uniform.

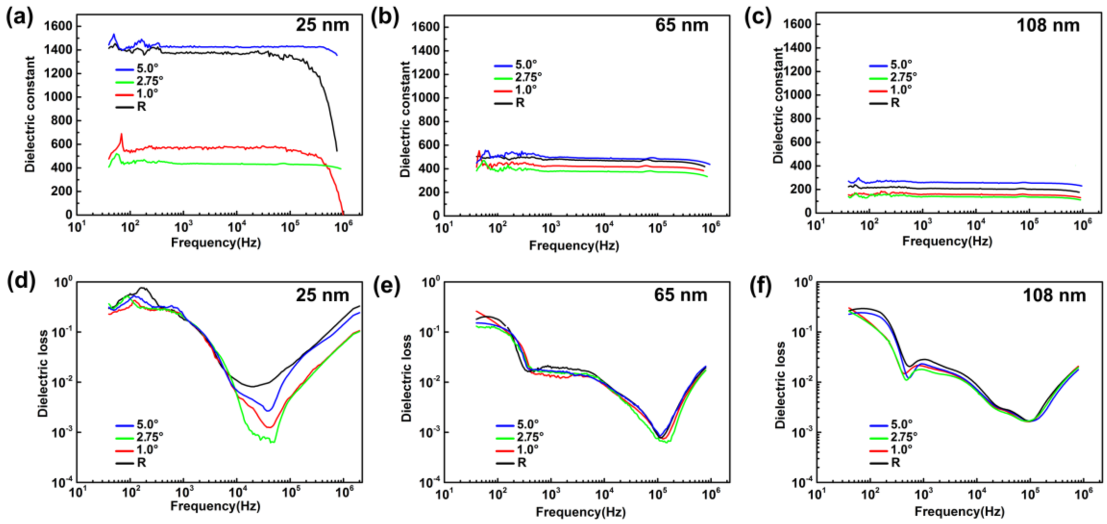

Figure S6. Dielectric constants and loss as a function of frequency for films grown on vicinal substrates.

Agilent 4294A Precision Impedance Analyzer is employed to test the dielectric properties of different thickness with the frequencies from 40 Hz (minimum frequency) to 2 MHz. The results show that the effect of vicinal substrates on the structure of CCTO film is attenuated in a thicker film. The modulation on CCTO films dielectric properties by the vicinal substrates decreased with the increase of the thickness, which should be attributed to the strain relaxation.
